# Reengaging Rural Research Participants After Two Decades: The NC Life Study’s Recruitment Challenges in an Era of Funding Uncertainty

**DOI:** 10.1080/08964289.2026.2653521

**Published:** 2026-10-21

**Authors:** Emily Forrest Hutchens, Marina Pearsall, Robert Faris, Alberto Valido Delgado, Liann Tucker, Nisha Gottfredson O’Shea, Tamara Taggart

**Affiliations:** a Department of Health Behavior, University of North Carolina at Chapel Hill; b Center for Behavioral Health and Well-being Research, RTI International; c Department of Sociology, University of California; d School of Education, University of North Carolina at Chapel Hill; e Department of Urban Global Public Health, Rutgers University; f Department of Social and Behavioral Sciences, Yale University

**Keywords:** adolescent health, health behavior, rural health, structural racism and discrimination

## Abstract

The North Carolina Life (NC Life, R01DA056264, MPI: O’Shea and Taggart) study is a 20-year follow-up to a groundbreaking adolescent study (R01DA013459, PI: Ennett), which examined the health behaviors of over 8,000 racially diverse adolescents in rural North Carolina communities during the early 2000s. This extension study aimed to leverage the initial R01 NIH investment by investigating health trajectories from adolescence through emerging adulthood, with a particular focus on how exposure to neighborhood-, health system-, social network-, and school-level structural racism influences these pathways. In April 2025, with one year of funding remaining, NC Life’s NIH support was terminated because it “no longer effectuates agency priorities.” This termination occurred after a considerable amount of time, effort, and funding had been invested into the complex process of re-contacting and enrolling thousands of rural participants, many of whom had relocated, changed contact information, or had no memory of their original study participation some twenty years prior. This paper presents an analysis of our recruitment methodology and outcomes including a variety of dynamic active and passive recruitment strategies to reengage research-hesitant rural communities. We conclude by detailing the cost of what has been lost by funding termination, reinstatement, and overall uncertainty. Our findings offer critical insights for the growing number of studies that may face similar reengagement challenges following termination or other extended study interruptions. The premature termination of NC Life represents a lost opportunity to advance understanding of how structural racism shapes health outcomes across the life course in understudied rural populations.

## PUBLIC HEALTH SIGNIFICANCE

This work illustrates the substantial losses incurred by federal funding uncertainty, including the disruption of community trust in the research process and institutions and the development of novel databases which facilitate our understanding of structural drivers of health in understudied communities.

## Introduction

Racial health inequities persist in the United States despite advances in disease prevention and healthcare. These inequities are driven by structural racism and discrimination (SRD), the unequal distribution of power and resources throughout interlocking social and economic systems.^1–3^ Additionally, health inequities related to SRD may be magnified in areas with additional disadvantage, such as rural and high poverty communities.^4–7^

Trust between public health institutions, researchers, and the public has been a topic of significant discussion in recent years.^8^ Trust between research teams and communities is essential not only to ethical research practice within the context of a single study, but to the sustainment of long-term perceptions of research and trust in research institutions and public health itself. These responsibilities are increasingly important among communities which have been excluded from the benefits of research development and the expansion of secondary education.^9–10^ Historical injustices in research practice, such as coerced participation, short-term engagement, and neglect of community priorities, have contributed to persistent mistrust of research among African American communities and rural populations alike.^11–12^

Because of the legacy of broken trust between communities and research institutions throughout the United States, African American and rural communities have reported that trustworthiness of researchers and institutions must precede trust building.^12–15^ Trustworthiness in practice has many iterations across varying study designs, all of which emphasize the importance of long-term investment in communities. In this manuscript, we describe a National Institutes of Health (NIH)-funded study in a racially diverse, rural community in central North Carolina (NC) which had its funding canceled during the volatile federal changes in April 2025.

The *North Carolina Life Study* (*NC Life*), funded by the National Institutes of Health (NIH) in 2022, is an extension of a seven-wave longitudinal accelerated cohort design study which began in 2002. The original study (hereto referred to as the adolescent study) involved a large (*n* = 8,196) and racially diverse (37.0% Black; 50.0% White) rural population of adolescents and included data about health and health behaviors as well as family life, school performance, social networks, and genetic information of a sub-sample. The adolescent study, also funded by the NIH, resulted in a robust dataset which was used in more than three dozen unique analyses and contributed significantly to modern understanding of adolescent health behaviors, including tobacco, drug, and alcohol use, bullying, intimate partner violence, peer violence, and mental health, and their contributing social factors. The *NC Life Study* extension aimed to leverage NIH’s initial investment by investigating health trajectories from adolescence to middle age, with a particular focus on how exposure to neighborhood-, health system-, social network-, and school-level SRD influence these pathways. *NC Life* was intended to create a longitudinal database spanning more than two decades, including more than 200 unique measures of chronic disease, health behaviors, social networks, and community resources, and was the first of its kind to allow for measurement of SRD indicators across the life course from adolescence to young adulthood in an understudied, racially diverse, rural population.

The *NC Life* team developed an innovative methodology to identify, contact, and reengage this research-hesitant population after nearly twenty years of disengagement from this research study. This approach leveraged archival data to perform multi-layered searches of public records and social media. A novel scoring system was developed to ensure accuracy in participant identities and streamline contact and linking to original data. Finally, a hybrid model of direct participant contact and community-based awareness campaigns were used to facilitate trust-building and improve reach. Early results demonstrate that even after two decades, meaningful reengagement was possible: thousands of participants were accurately identified and directly contacted, community partnerships were built to broaden awareness, and enrollment rates were positive.

In April 2025, *NC Life’s* NIH support was retroactively terminated, with funding ending in February 2025. The termination was justified under the assertion that: “Research programs based primarily on artificial and nonscientific categories, including amorphous equity objectives, are antithetical to the scientific inquiry, do nothing to expand our knowledge of living systems, provide low returns on investment, and ultimately do not enhance health, lengthen life, or reduce illness.” This termination occurred after considerable time, effort, and funding had been invested into the complex process of engaging participants who are rural, high poverty, and generally disengaged from and distrustful of research and higher education.^16^

Due to a case brought against the NIH by the American Public Health Association, funding for six months of the final project year was reinstated starting in August 2025. Although this funding provided our team with resources needed to close out the project, the termination damaged years of groundwork in ways that are yet to be fully understood, though three critical losses are already apparent. First, termination disrupted recruitment at a sensitive stage, halting momentum when community-level awareness was gaining traction and enrollment was accelerating. Second, it damaged the fragile trust between researchers, universities, and racially diverse rural communities already marked by justified skepticism of academic research. Third, it halted the creation of a novel, irreplaceable dataset that would have provided unprecedented insights into the health and social trajectories of a densely connected, racially diverse, rural population, and the impacts that SRD has on these trajectories. This paper documents the recruitment and reengagement strategies developed by the *NC Life* team, the time and resource investment necessary for this novel work, and the critical losses which sudden funding termination imposed on vital health disparities work.

## Methods

### Original study and contributions to science

The goal of the original adolescent study was to obtain rich measures of the social and environmental contexts to understand how these multi-level factors affect adolescent development across the middle- and high-school years. In Spring 2002, all public and charter middle schools within three North Carolina counties permitted researchers to administer school-wide surveys to 8,196 6^th^, 7^th^, and 8^th^ grade students with parental assent, excluding students who could not participate, either because they were English language learners or due to moderate or severe learning disabilities. In-school surveys were repeated in Fall 2002, Spring 2003, Fall 2003, Spring 2004, Fall 2004, and Fall 2005. Surveys were administered to students in public or charter high schools once students transitioned out of middle school, as long as they continued to attend school in one of the study counties. Additionally, a core sample of 1,500 students was selected for participation in the longitudinal parent surveys. This unique, accelerated design allowed researchers to observe development from 6^th^ to 12^th^ grade in only four years. Table 1 shows participant and school enrollment and grades collected during each study wave.

In line with the goal of clearly capturing the social and environmental contexts of adolescents, the surveys: asked students about their peer relationships, including bullying and victimization and dating violence; assessed use of alcohol, tobacco, and other drugs, as well as peer (dis)approval, familial models and parental beliefs about substance use, and consequences of use; asked about how connected students felt to their school, their teachers, and extracurricular activities; asked students to evaluate neighborhood safety and belongingness; and assessed family structure and parental warmth and monitoring; mental health, sensation-seeking, deviant behaviors, prosocial beliefs, religiosity, and grades in school.

The original adolescent study resulted in several dozen peer-reviewed manuscripts. Here, we briefly touch on a few contributions of this body of work.

Bidirectional friend, bully, and victim nominations between students within each of the 19 participating schools produced a wealth of longitudinal social network data which, in turn, generated novel information about the nature of social networks, as well as factors preceding or resulting from changes in social networks. This knowledge has contributed substantially to how bullying behavior is understood,^17–18^ when and why it occurs,^19–22^ its consequences for both bullies and victims over time,^23–25^ and the conditions under which bullying behavior develops into dating violence perpetration.^26–27^

Social network data also enabled the team to understand nuanced social dynamics in peer selection and influence in adolescent substance use^28–32^ and the complex role of parents as unwitting models or enablers of adolescent substance use.^33^ The knowledge generated from these studies led directly to the development of novel preventive interventions targeting adolescents and their parents, including interventions to prevent dating violence^34^ and early onset alcohol use.^35,36^

Data from the adolescent surveys also highlighted a discrepancy in the explanatory power of our statistical models to explain substance use, internalizing symptoms, deviant behavior, and longer-term conviction outcomes for Black participants compared with white participants. Despite its rich self-report measures, the adolescent survey did not adequately capture *structural* factors that are essential for explaining Black-white disparities in health outcomes.

### Participant identification

As *NC Life* sought to connect data from adolescence into adulthood in order to characterize the influence of SRD on health trajectories, the accurate identification, re-contact, and reenrollllment of as many original study participants as possible was necessary. While many methods for recontacting ongoing study participants have been proposed, few provide guidance for the unique challenges and advantages of recontacting participants after a long period of disengagement in cohort studies.

A first challenge was the extended length of time between contact, in this case, nearly 20 years. A second challenge was the nature of the original study participation. Because the original survey was distributed and collected as part of the normal school day, by students’ usual teachers and without much additional emphasis, we suspected and later found that most original study participants did not recall being part of the original study at all.

A primary advantage of connecting these two studies was the availability of social network data combined with the rural setting and racially diverse population. Robust social network data available from the adolescent study, despite being focused on adolescence, allows for the potential to recruit *via* known networks. Additionally, as nearly all students across entire school districts in these rural counties were enrolled in the adolescent study, these networks are unique in that they are nearly complete. The authors were not able to locate any guidance regarding study recruitment procedures for this situation and therefore developed a novel method for conducting similar future work.

We used a three-phase recruitment methodology that included identification, data extraction, and contact. A detailed identification, extraction, verification, and contact process was created by study staff in Spring 2022 and revised throughout the course of the study, as challenges were discovered and addressed. Between August 2022 through December 2024, a total of 107 student research assistants implemented the identification phase. Next, in January 2024, we began the contact and enrollment phase, which continued until April 2025.

Research assistant recruitment and training was conducted on a semester basis, including summer session. Orienting new research assistants to the study and training them in the identification and extraction systems and processes took approximately two weeks per new cohort. Training new and continuing research assistants in the contact process for both phone contact and in-person community outreach took approximately three weeks, with rigorous training in clear communication about the study and consent procedures. For a sub-group of research assistants who performed only social media contact, training took approximately two weeks. Research assistant turnover rates varied between 30.0% and 80.0% per semester. Therefore, training was re-conducted each semester with all new research assistants and with all research assistants when moving between phases. Thus, the time and labor required to hire, train, and supervise a large number of research assistants was significant but necessary investment in order to engage directly with community members at this scale.

Available data from the adolescent study for identification of current study subjects included the following: name during adolescence, date of birth, landline phone number of childhood home(s), childhood home address(es), parent or guardian name(s), school name, and approximate graduation class. Final graduation year was not available because the adolescent study concluded when the oldest cohort was in its senior year. As with all self-reported research data, the original adolescent study data has inconsistencies related to spelling, data entry, and accuracy across study waves. Data were first cleaned and collated across waves to ensure consistent data for each participant. All original study data were uploaded into the Ragic database (a secure, cloud-based data platform) which created a profile for each participant. Each participant profile includes original study data used for identification as well as information generated from the identification process.

The research team first identified present-day participants using publicly available data sources, including online background search tools, voter registration records (which are public in North Carolina), obituaries, newspapers and online news sites, and social media, including Facebook, Twitter, Instagram, and LinkedIn. Participants who could not be identified through publicly available data sources were further investigated by a secondary team who conducted a supplementary search using physical and archival media, including yearbooks, Facebook groups, and local media. Documentation of publicly available information for each participant was stored in the Ragic database.

### Quality scoring

After the first full search was conducted, data were extracted by a second research assistant for each participant profile, and each participant’s information matching between original study data and gathered identity information was scored for quality on a scale of 0 to 4. This provided the opportunity for a secondary investigation of each case, an additional step in promoting accuracy in matching between original participants and present-day participants and provided a framework for the research team to prioritize which participants to reach out to during later recruitment. Quality Scoring was conducted based on the amount of information which matched between the original study data and publicly available data, as well as between publicly available data sources. Participants for whom a high level of confidence was established in matching between the adolescent study data and publicly available data received a 4, while participants for whom fewer data points matched received a 3, and so on, until participants for whom no publicly available data was found received a 0.

Participants were prioritized for direct contact in descending order based on their Quality Score assigned during the Identification phase, wherein participants with the highest confidence of matching between the adolescent study data and publicly available data were contacted first. Additionally, repeated direct contact was attempted with participants who were identified as being highly connected in their school social network (in the top quintile of social network centrality) based on original study data. These participants were also contacted directly over social media through identified Facebook accounts.

### Community awareness and engagement

Broader community awareness campaigns were also conducted in addition to direct contact, including ads on Facebook and Google, posts on Facebook groups for local communities and schools, and physical and online flyers posted and distributed at local businesses, churches, schools, and community events. These campaigns were largely focused in areas where targeted participants were likely to congregate and work, according to their age and long-term residence, such as elementary schools, which tend to employ a large amount of teachers who were A) in the middle-adulthood age range, B) raised in the community, and C) well-connected to others who might be eligible, such as parents of elementary-age children. Community awareness campaigns included a referral program wherein any individual who referred eligible participants based on general information such as school and graduating class received a $25 gift card. A community ambassador awareness program was designed in late 2024 which engaged participants who were highly embedded in their community, as identified *via* adolescent study social network data as well as present study data. Implementation of the ambassador program was planned for Spring 2025 but was postponed due to study termination. At the time of study termination, three community ambassadors had been recruited into the program and were informed that study activities could no longer continue. In September 2025, the study team reached back out to ambassadors and were able to reenrollll two ambassadors.

Participants for whom fewer than two data points matched between the adolescent study and the screener were elevated to manual review by a research team member, who checked data by hand to confirm or deny eligibility. Screener completers who had some data correct but some mismatches were contacted directly *via* phone or email to further confirm identity and eligibility. Those confirmed as eligible then provided informed consent before receiving their survey link.

Participants who were identified as eligible were invited to complete an online survey that would take approximately one hour to complete and received a $50 gift card upon completion. Participants could leave and return to the survey without losing their place and all responses were automatically saved. The survey included questions about community and family life, employment, health status, health behaviors such as substance use and healthcare utilization, community connectedness, and experiences of criminalization and SRD.

## Results

Research assistants successfully traced 7,488 (91.4%) of 8,196 eligibles (93.0% of original study participants). Of those, we achieved medium to high confidence that we successfully matched public records identifiers to our respondents in more than three-quarters (76.7%) of cases (*n* = 5,740). The majority of those traced had never left North Carolina, and approximately two-thirds currently resided in or near the original study counties. However, 668 could not be traced, even using full names, previous addresses, and birth dates. This raises a question of both theoretical and practical significance: who disappears from public view? To answer this question, researchers estimated a logistic regression on disappearance—defined as the 7.0% of cases for whom we could find no records at all. We controlled for the frequency with which their first and last names appear, according to the US Census. Controlling for name frequency, Latinx and white youth were significantly more likely to vanish from public records. Those who lived in more populated areas, with lower centrality in the school’s social network, and who moved residences during middle or high school were also more likely to disappear from public records. We will use these results in the development of sample weights to mitigate known biases in future analyses—presuming that NC Life collects sufficient follow up data for analysis.

The Quality Scoring process was directly tied to final study participation, as those who were ranked higher in the Quality Score were increasingly likely to complete the survey. See Table 2 for quality scoring among *NC Life Study* participants.

Participants were contacted directly to participate in NC Life through a variety of strategies, including cold calling, automated voicemail messages, text messages, emails, social media, and physical postcards and letters. Survey respondents were primarily women, White, highly educated (holding a Bachelor’s Degree or higher), and from households reporting income of more than $50,000 per year. See Table 3 for NC Life participant demographics as of February 2025. See Figures 1 and 2 for visualizations of enrolled participants’ race/ethnicity, sex at birth and educational attainment.

## Discussion

As part of the multi-tiered recruitment strategy, researchers frequently visited the three study counties, making canvasing visits to local businesses, libraries, schools, churches, and community colleges. Researchers spoke at community meetings and hosted booths at local fairs, parades, and high school football games. Principals at schools in the three study counties were contacted *via* mailed letter, phone, and email. In the largest county, the study team received district-level approval to distribute study recruitment flyers and delivered flyers to all elementary schools for distribution to staff members and parents of kindergarten and first grade-aged students. Fifty-two churches were identified and contacted, primarily through email and phone. Flyers were posted and delivered to a variety of organizations and businesses, including local businesses, nonprofit organizations, public libraries, community colleges, and city administrators. Staff members held booths at several community events, including community meetings, a local Fall festival, and football games. Postcards with study information were mailed to parents of previous study participants, individuals who were identified as living outside North Carolina, and parents whose children had been identified as “highly connected” during high school. As these activities continued, researchers heard increasing familiarity with the study from the community at large. When those who were eligible but had not yet enrolled in the study were asked, informally, about their participation, these potential participants reported that they were distrustful of cold phone calls, texts, and emails, which often involved either scam attempts or political messaging, which was rampant in the years leading up to the 2024 Presidential Election.

Community trust between researchers and potential participants was fostered not only during discussion of the study itself, but through richer connection at these events. When a research team member was invited to speak at a women’s group meeting at one study site, discussions about the study were followed by further discussion about health and healthcare access in the rural area. When the conversation turned to motherhood, community members shared their stories of difficulty with fertility, raising children, and career advancement and the researcher did the same, comparing experiences among those in the group who worked at home, were military spouses, and were working mothers. Following this conversation, the group members had more follow-up questions about the landscape and process of university-based health research. This interaction transcended study recruitment, fostering meaningful bonds between a representative of both the university and scientific research as a whole and a community which had limited interaction with either.

In order to capitalize on the in-person trust building established during researcher outreach, the research team developed a Community Ambassador program in early 2025. Potential Ambassadors were NC Life study participants who were highly connected and who were active members of their community, such as teachers, religious leaders, hairdressers or barbers, or parents of school-age children. At the time of funding termination, three potential Ambassadors had been recruited and interviewed by the research team and were preparing to begin outreach.

A primary advantage of connecting these two studies was the availability of social network data combined with the rural setting and racially diverse population. Robust social network data available from the adolescent study, despite being focused on adolescence, allows for the potential to recruit *via* known networks. However, these close social ties similarly allow distrust toward an institution or research program to fester and expand.

The termination of this funding has resulted in damaging the trust between these rural, isolated communities and both the university and public health research at large.

Racial inequities in health persist despite advances in health promotion, innovation, and increased access to health services. It is theorized that these inequities are driven by SRD, which reinforces unequal distributions of power and resources among Black and White communities, resulting in unequal distribution among neighborhoods, schools, and families.^1–3^ These inequities manifest differently throughout the life course,^37^ yet there is not currently a longitudinal dataset that was designed with the express purpose of investigating the potential lifelong and cumulative effects of SRD. The termination of *NC Life* impaired the creation of what would be one of the most unique and valuable longitudinal datasets on rural health, health behaviors, and socialization in the United States. The project stood alone in its capacity to connect adolescent and adult data across a twenty-year span within a racially diverse, tightly interconnected, and largely rural population.

The original adolescent study enrolled 99.0% of middle and high school students at the study sites with an IRB-approved passive consent process (IRB #22-1502, UNC Chapel Hill), by which parents or students could opt out of the study. Today, most studies of adolescents require participants and their parents to opt-in *via* active consent, which dramatically reduces response rates. This introduces biased samples, and particularly degrades social network data, which rely on high completion rates from all actors in the network. The adolescent study’s near-complete social network data provided a rare opportunity to study how social network connections and density influence nuanced adolescent behaviors, from substance use to bullying and intimate partner violence. The unique opportunity to investigate how these networks continue to influence health and health behaviors into adulthood and namely, how these outcomes can help to characterize the influences of structural racism, have now been compromised.

*NC Life* would have provided one of the only datasets in the U.S. capable of linking adolescent networks and environments to adult outcomes in a rural context, and one of the very few longitudinal datasets in which Black and White rural residents were both well-represented. As rural health data are often geographically scattered, demographically homogeneous, and cross-sectional, *NC Life*, by contrast, was positioned to allow investigation into how long-term exposures within schools, families, and communities accumulate to influence health disparities. These data would have been invaluable not only for behavioral health researchers, but for sociologists, economists, and public health practitioners seeking to understand how community structure and racial inequity intersect in shaping long-term outcomes.

## Conclusions

The *NC Life Study* demonstrates the feasibility of reengaging a research-resistant and understudied rural community after more than two decades of disengagement. However, the successful tracking and identification efforts and active community engagement requires extensive time, planning, and human resources. Progress thus far indicates that sustained investment could yield continuing survey completion with the ultimate goal of building a robust longitudinal dataset which builds on previous NIH investments. The premature termination of NIH funding interrupted this progress, disrupted community partnerships, and halted the development of a unique data resource which would provide opportunity to investigate how structural racism and discrimination function in this unique community. Ongoing support for this work is essential to maintain trust, ensure data integrity, and advance scientific knowledge about health disparities.

## Figures and Tables

**Figure 1.**
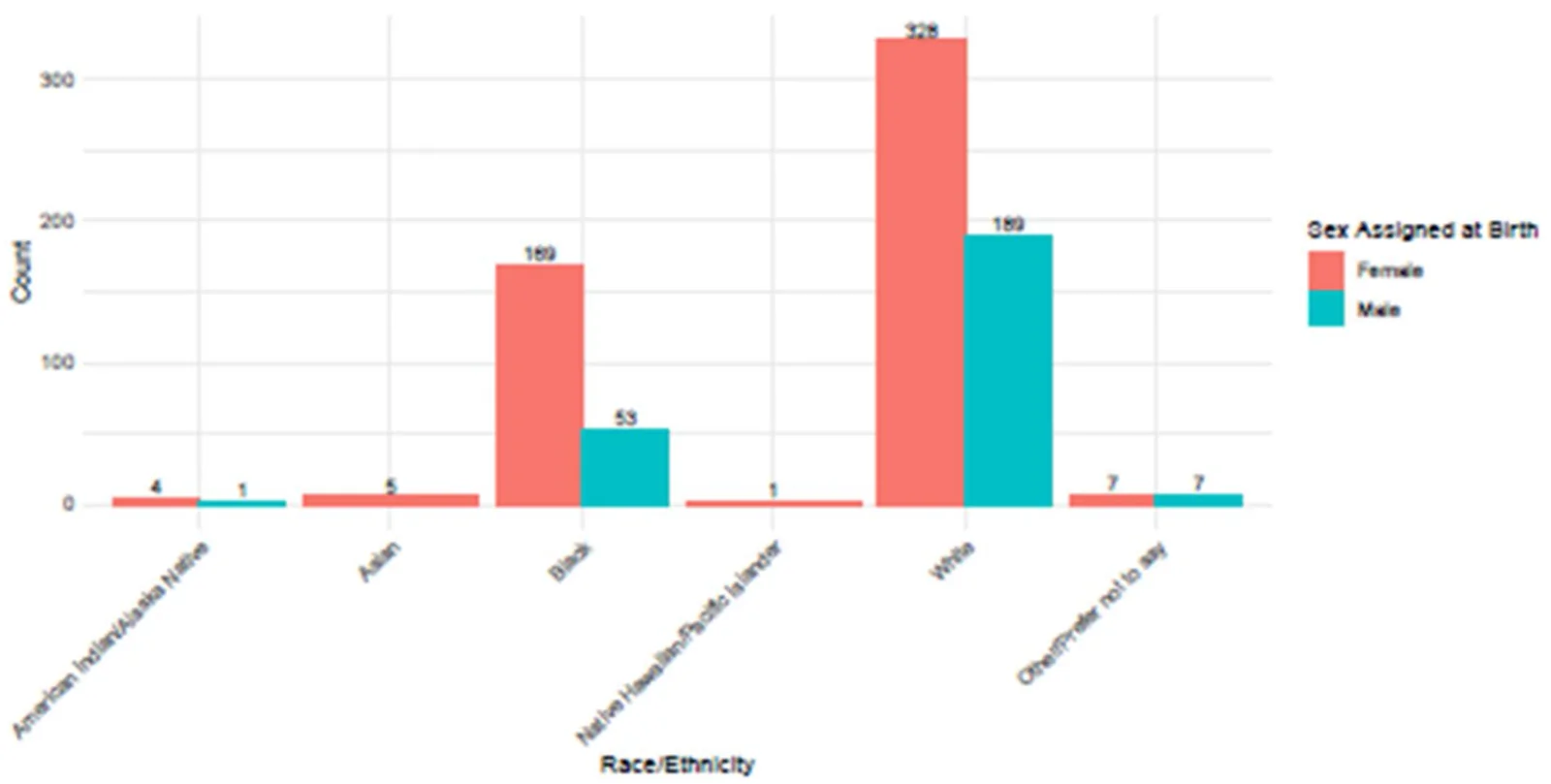
Race/ethnicity and sex of enrolled participants through February 2025.

**Figure 2.**
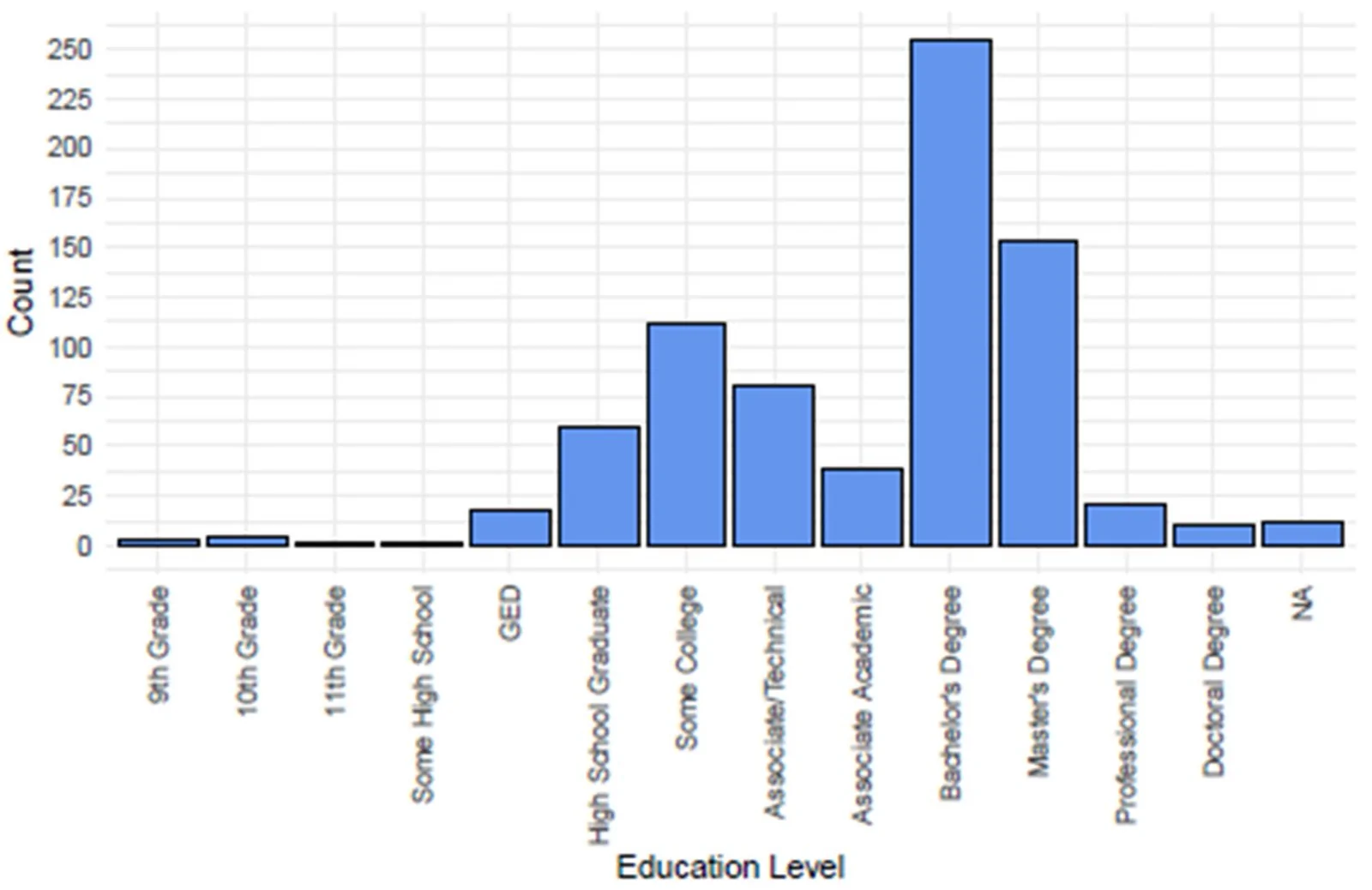
Highest level of education of enrolled participants through February 2025.

**Table 1.** Adolescent study waves.

	Data collection wave						
	1	2	3	4	5	6	7
Date	Spring ‘02	Fall ‘02	Spring ‘03	Fall ‘03	Spring ‘04	Fall ‘04	Fall ‘05
School system N	3	3	3	3	3	2	2
Grades	6, 7, 8	7, 8, 9	7, 8, 9	8, 9, 10	8, 9, 10	9, 10, 11	10, 11, 12
School N (middle/high/alternative)	13 (10/0/3)	19 (10/6/3)	19 (10/6/3)	19 (10/6/3)	19 (10/6/3)	5 (0/3/2)	5 (0/3/2)
Peer network N	33	29	29	19	19	5	5
Adolescent N (response rate)	5220 (88.4%)	5060 (81.3%)	5059 (80.9%)	5017 (79.1%)	4676 (76.0%)	2419 (75.1%)	2131 (72.8%)

**Table 2.** Quality scoring *via* identification process.

Quality score	Quality score definition	*N* participants	% of original study population
0	No information found: This subject does not appear in any of the search tools.	701	8.6%
1	One data item matched: Only one data item could be matched between the original adolescent study data and any one of the search tools.	1,755	21.4%
2	Limited information matched: Two data items could be matched between the original adolescent study data and any one of the search tools OR only one data item was matched between the adolescent study data and across two search tools (e.g., mother’s matched between the adolescent study data, Family Tree, and Facebook) OR one data item matched between the adolescent study and one search tool and a different data item matched between the adolescent and another search tool. Conflicting information may be found between search tools.	1,184	14.4%
3	Moderate information matched: Three data items could be matched between original adolescent study data and any of the search tools OR two data items could be matched between the adolescent study and across multiple search tools. Conflicting information may be found between search tools.	2,159	26.3%
4	High degree of information matched: Four or more pieces of information could be matched between original adolescent study data and any of the search tools.	2,397	29.2%

**Table 3.** Participant demographics summary through February 2025.

Demographic	Count	Percentage
**Sex Assigned at Birth**		
Female	514	66.8%
Male	250	32.5%
**Race/Ethnicity**		
American Indian/Alaska Native	5	0.7%
Asian	5	0.7%
Black	222	28.9%
Native Hawaiian/Pacific Islander	1	0.1%
Other/Prefer not to say	19	2.5%
White	517	67.2%
**Education**		
9th Grade	3	0.4%
10th Grade	4	0.5%
11th Grade	2	0.3%
12th Grade/High School	2	0.3%
GED	18	2.3%
High School Graduate	60	7.8%
Some College	111	14.4%
Associate Technical	86	10.4%
Associate Academic	38	4.9%
Bachelor’s Degree	255	33.2%
Master’s Degree	153	19.9%
Professional Degree	21	2.7%
Doctoral Degree	10	1.3%
**Household Income**		
Less than $10,000	31	4.0%
$10,000-$14,999	19	2.5%
$15,000-$24,999	37	4.8%
$25,000-$34,999	39	5.1%
$35,000-$49,999	72	9.4%
$50,000-$74,999	129	16.8%
$75,000-$99,999	113	14.7%
$100,000-$149,999	154	20.0%
$150,000-$199,999	86	11.2%
$200,000 or more	69	9.0%
Don’t know	9	1.2%
